# Phenotypic Diversity and Altered Environmental Plasticity in *Arabidopsis thaliana* with Reduced Hsp90 Levels

**DOI:** 10.1371/journal.pone.0000648

**Published:** 2007-07-25

**Authors:** Todd A. Sangster, Adam Bahrami, Amity Wilczek, Etsuko Watanabe, Kurt Schellenberg, Catherine McLellan, Alicia Kelley, Sek Won Kong, Christine Queitsch, Susan Lindquist

**Affiliations:** 1 Committee on Genetics, University of Chicago, Chicago, Illinois, United States of America; 2 Whitehead Institute for Biomedical Research, Cambridge, Massachusetts, United States of America; 3 Department of Organismic and Evolutionary Biology, Harvard University, Cambridge, Massachusetts, United States of America; 4 FAS Center for Systems Biology, Harvard University, Cambridge, Massachusetts, United States of America; 5 Informatics Program, Children's Hospital Boston, Boston, Massachusetts, United States of America; University of Chicago, United States of America

## Abstract

The molecular chaperone HSP90 aids the maturation of a diverse but select set of metastable protein clients, many of which are key to a variety of signal transduction pathways. HSP90 function has been best investigated in animal and fungal systems, where inhibition of the chaperone has exceptionally diverse effects, ranging from reversing oncogenic transformation to preventing the acquisition of drug resistance. Inhibition of HSP90 in the model plant *Arabidopsis thaliana* uncovers novel morphologies dependent on normally cryptic genetic variation and increases stochastic variation inherent to developmental processes. The biochemical activity of HSP90 is strictly conserved between animals and plants. However, the substrates and pathways dependent on HSP90 in plants are poorly understood. Progress has been impeded by the necessity of reliance on light-sensitive HSP90 inhibitors due to redundancy in the *A. thaliana* HSP90 gene family. Here we present phenotypic and genome-wide expression analyses of *A. thaliana* with constitutively reduced HSP90 levels achieved by RNAi targeting. HSP90 reduction affects a variety of quantitative life-history traits, including flowering time and total seed set, increases morphological diversity, and decreases the developmental stability of repeated characters. Several morphologies are synergistically affected by HSP90 and growth temperature. Genome-wide expression analyses also suggest a central role for HSP90 in the genesis and maintenance of plastic responses. The expression results are substantiated by examination of the response of HSP90-reduced plants to attack by caterpillars of the generalist herbivore *Trichoplusia ni*. HSP90 reduction potentiates a more robust herbivore defense response. In sum, we propose that HSP90 exerts global effects on the environmental responsiveness of plants to many different stimuli. The comprehensive set of HSP90-reduced lines described here is a vital instrument to further examine the role of HSP90 as a central interface between organism, development, and environment.

## Introduction

The essential eukaryotic chaperone HSP90 facilitates the maturation of a variety of metastable protein substrates, many of which are central regulators of biological circuits [1]–[3]. Due to HSP90's central position in numerous pathways regulating growth and development, the chaperone has emerged as a principal focus of investigation in diverse fields. For example, as key proteins controlling cell proliferation depend on HSP90, it is an intensively investigated target for anti-cancer therapies [4]. More broadly, HSP90 activity has been shown to have widespread effects on the genesis of phenotypic diversity. HSP90 can suppress the phenotypic consequences of existing genetic polymorphisms [5], [6], affect epigenetically inherited phenotypes [7], and facilitate the spread of novel mutations [8]. Further, reduced HSP90 function can increase the probability that stochastic events inherent to development result in phenotypic differences [5], [9].

Molecular chaperones are proteins which can modulate the folding of a variety of other polypeptides without permanent alteration to themselves [3]. HSP90 is distinct among molecular chaperones in its functionally diverse but numerically restricted set of substrates (client proteins), which tend to share an inherent conformational instability. HSP90's interactions with certain mammalian hormone receptors, such as the glucocorticoid receptor [10], [11], serve as a paradigm for its molecular function. HSP90 maintains such substrates in a signaling competent state, enabling them to receive upstream signals, such as hormone binding. Substrates then typically undergo a conformational change, thereby propagating signal transduction to downstream effectors [2], [3].

HSP90 function and substrates have been best investigated in mammalian and fungal systems [12]. However, the biochemical function of HSP90—to promote the maturation of metastable client proteins—is conserved in plants, as illustrated by the fact that obligately HSP90-dependent mammalian steroid hormone receptors are functional in the plant cytoplasm, even though plants lack any homologous receptors [13].

In the plant *Arabidopsis thaliana*, HSP90 homologs are encoded by seven different genetic loci. Of these, one is expressed in the endoplasmic reticulum (*HSP90.7*), one in the mitochondrion (*HSP90.6*), one in the chloroplast (*HSP90.5*), and four in the cytosol. The gene encoding one cytosolic protein (*HSP90.1*/At5g52640) is highly stress-inducible, whereas the other three (*HSP90.2*/At5g56030, *HSP90.3*/At5g56010, and *HSP90.4*/At5g56000) are constitutively expressed and are the products of very recent duplication events [14], [15]. Two of the organelle specific HSP90s, have been shown to affect plant development. A choloroplast HSP90 mutant, *cr88*, alters responses to red light, chlorate resistance, and delays chloroplast development [16]. An endoplasmic reticulum HSP90 mutant, *shepherd*, affects apical meristem maintenance, presumably due to the failure to adequately chaperone the *CLAVATA1/2* complex [17].

The functions of the cytosolic HSP90s are not well understood; mutations in these genes have only been phenotypically connected to increased sensitivity to biotrophic parasites, primarily microbial pathogens and viruses [18]–[24]. The pathways that HSP90 is known to influence share dependency on R-proteins, which facilitate pathogen recognition and initiate downstream defense responses, such as localized cell death (hypersensitive response) and induced systemic resistance [25]. Several groups have demonstrated the essential role of HSP90 in maintaining the stability of several R-proteins [18], [19], [21], [26], [27]. If HSP90 function is missing, these R-proteins are rapidly degraded, akin to observations with HSP90 substrates in other systems [28], presumably leading to pathogen sensitivity.

A point mutation (*hsp90.2-3*) in the ATP-binding domain of one of the constitutive cytosolic HSP90s, HSP90.2, renders the plant sensitive to pathogens [19]. However, a null mutation in *HSP90.2* failed to display the same phenotype. Point mutations in similar positions to *hsp90.2-3* in yeast HSP90 proteins have been analyzed biochemically and found to prevent either ATP binding or hydrolysis [29], [30], thereby interfering with substrate release [31]. Therefore, the likely substrate-trapping properties of the *hsp90.2-3* mutation may result in some dominant characteristics and stronger phenotypes when compared to a corresponding null mutation.

Neither the *HSP90.2* null mutation nor *hsp90.2-3* displayed common obvious morphological abnormalities in previous work [19], suggesting redundancy between different cytosolic isoforms. Inhibition of HSP90 activity with geldanamycin (GDA), an inhibitor that is specific to the HSP90 family of proteins, resulted in dramatically increased phenotypic variation in ten day old seedlings, affecting leaf shape and color, hypocotyl elongation, and gravitropism responses among others [5]. As GDA is light-sensitive, HSP90-dependent phenotypes could only be analyzed at the seedling stage.

To comprehensively investigate the phenotypic consequences of reduced cytosolic HSP90 function, we obtained lines with transfer DNA (T-DNA) insertions in *HSP90.1, HSP90.2*, or *HSP90.3*, representing null mutations in the respective isoforms [32]. As *HSP90.2, HSP90.3*, and *HSP90.4* are located within 12000 base pairs of each other [33], the construction of double mutants for constitutive isoforms is extremely difficult. Therefore, we decided to target the cytosolic HSP90s with a RNA interference (RNAi) based approach, creating a set of lines with reductions in the levels of several cytosolic isoforms. Herein, we describe the phenotypic and molecular characterization of all of these HSP90-reduced lines.

## Results

### Construction of HSP90-Reduced Lines

**Figure 1:**
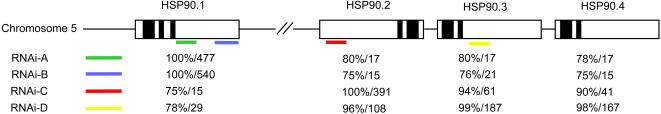
Targeting HSP90 with double-strand RNA constructs. The four cytosolic HSP90s are depicted in their genomic context. Introns are in black, exons in white. Regions cloned to create the double-strand RNA (RNAi) constructs are indicated in green (RNAi-A), blue (RNAi-B), red (RNAi-C), and yellow (RNAi-D) bars. Below each isoform the percent identity and the longest consecutive number of bases of perfect identity to the cloned region are noted for each construct.

To interfere with the expression of multiple cytosolic HSP90 isoforms, we cloned four regions of HSP90 with differing sequence identity across the four cytosolic isoforms. Construction of double-strand RNA producing vectors (Fig. 1) and transformation into the *A. thaliana* Col-0 accession were performed as described in [34] with the modifications detailed in the Methods. Control lines were created with an identical vector lacking any HSP90 sequence. The sequence-verified constructs contained a kanamycin resistance cassette, allowing screening for positive transformants in the T_1_ generation. In total, vectors containing four different regions of HSP90 were tested. Screening of ∼50,000 total seeds for each construct from three independent transformations yielded at least ten transgenic lines for three of the constructs. No transformants were obtained for the fourth construct (RNAi-D), which had the highest likelihood of interfering with expression of all four isoforms. Given the success with the other constructs, it appears likely that HSP90-reduction caused by the RNAi-D construct is severe enough to cause lethality under standard growth conditions.

Positive transformants were self-propagated and analyzed for kanamycin resistance frequency in the T_2_; lines with frequencies consistent with segregation of a single insert were selected for further analysis. Kanamycin resistance was used to isolate true-breeding lines for the constructs from the T_3_ generation. Of these, five lines, including three containing HSP90-RNAi construct A and one containing each of constructs B or C, were chosen for subsequent experiments, along with three independent control lines. Insert locations for most lines were determined by standard methods [35]; none of the isolated insert locations were within a coding region (Methods).

As a complementary resource, we obtained lines with single T-DNA insertions in the *HSP90.1*, *HSP90.2*, and *HSP90.3* isoforms (HSP90-TDNA lines) [32] from the Arabidopsis Biological Resource Center. Lines homozygous for each insert were isolated by PCR screening.

### Assessment of HSP90-Reduction

Quantitative Western blotting was used to confirm that HSP90 protein levels are constitutively decreased under ambient conditions in the HSP90-RNAi and HSP90-TDNA lines. The antibody employed should recognize all four cytosolic HSP90s [36]. All lines exhibited significantly reduced HSP90 levels except for the HSP90.1-TDNA line (Table 1). As HSP90.1 is constitutively expressed at low levels, a null mutant in this isoform would not be expected to exhibit HSP90 reduction under ambient conditions.

**Table 1:** HSP90 levels in HSP90-RNAi and HSP90-TDNA lines.

Genotype	Quantitative Western Blot		RT-PCR Cycle Number	
	Mean	Standard Deviation	Mean	Standard Deviation
Control-1 T_3_	102	7.1	n.d.	n.d.
Control-2 T_3_	92	7.1	19.6	0.39
Control-2 T_4_	n.d.	n.d.	19.8	0.59
Control-3 T_3_	106	6.0	n.d.	n.d.
RNAi-A1 T_3_	68*	26.9	21.2**	0.25
RNAi-A2 T_3_	70**	12.5	20.9**	0.18
RNAi-A2 T_4_	n.d.	n.d.	20.9**	0.37
RNAi-A3 T_3_	66**	14.4	20.8**	0.27
RNAi-B1 T_3_	52**	12.3	22.0**	0.07
RNAi-C1 T_3_	69**	7.8	n.d	n.d
Control (Col-0)	100	10.1	n.d	n.d
*hsp90-1*	115	12.5	n.d	n.d
*hsp90-2*	50**	12.0	n.d	n.d
*hsp90-3*	43**	22.2	n.d	n.d

** significant at p<0.01

* significant at p<0.05 (t-test)

Protein for quantitative Western blots was extracted from plants grown under ambient conditions. The HSP90 antibody used should recognize all four cytosolic isoforms. Values are normalized such that the average of the control lines is 100; HSP90-RNAi and HSP90-TDNA lines are normalized separately. RNA for RT-PCR was extracted from plants heat shocked at 37°C for two hours. RT-PCR primers recognize only HSP90-1. n.d., not determined.

Consistent with these results, quantitative RT-PCR of RNA derived from seedlings which had undergone a brief heat-shock revealed significantly lower levels of HSP90 RNA in the HSP90-RNAi lines (Table 1). The reduction is reproducibly at least two-fold (one cycle) in all lines. The primers used in this reaction amplify *HSP90.1* only. Importantly, the reduction in HSP90 levels also appears to be stable across generations (Table 1).

Further confirmation that HSP90 levels are reduced in both HSP90-RNAi and HSP90-TDNA lines was derived from genome-wide expression analyses. All HSP90-RNAi and HSP90-TDNA lines were analyzed, along with the *hsp90.2-3* mutant. Ten 14 day old seedlings per genotype were pooled for each sample, and replicate samples were analyzed with the Affymetrix ATH-1 microarray. All array data may be found in the Gene Expression Omnibus under accession number GSE7796.

Standard methods were used to determine which genes were differentially expressed between each HSP90-reduced line and the appropriate control (Methods). Our expression data confirmed that the cytosolic HSP90 isoforms tended to be reduced between 1.5 and 2 fold in a statistically significant manner in all HSP90-RNAi lines; individual isoforms were reduced to different extents in different lines. The magnitude of the reduction of total HSP90 RNA levels in each line is consistent with the RT-PCR data. *HSP90.1* and *HSP90.2* transcripts were significantly reduced to nearly undetectable levels in the respective HSP90-TDNA lines. The probes on the array cannot differentiate between *HSP90.3* and *HSP90.4*; the combined transcript levels of these genes were significantly reduced six fold in the HSP90.3-TDNA line. Note that in the HSP90.2-TDNA and *hsp90.2-3* mutants, *HSP90.1* levels were two-fold upregulated, indicating cross-regulation between different isoforms. This subtle increase in *HSP90.1* expression is likely due to a slight induction of the heat-stress response in the HSP90-TDNA lines, as other heat-stress markers such as *HSP101* show similar levels of induction. The induction of the heat-stress response by reduced levels of HSP90 has been previously observed in other systems [37], [38]. Such induction was not observed in any of the HSP90-RNAi lines, likely due to their lower levels of total HSP90 reduction.

### Increased Phenotypic Diversity in HSP90-Reduced Seedlings

**Figure 2:**
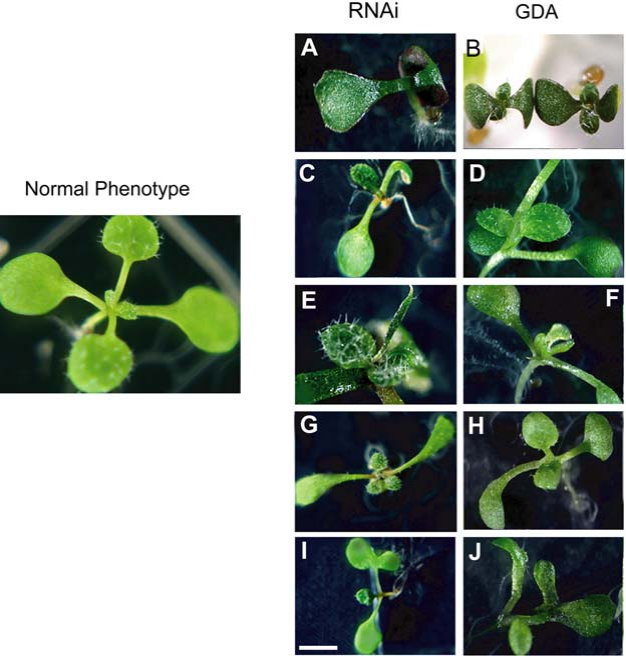
Similar morphological phenotypes of seedlings with reduced HSP90 function by RNAi or pharmacological means (GDA). (a) and (b): purple pigment accumulation; (c) and (d): organ number defect; (e) and (f): narrowly-shaped deformed true leaves; (g) and (h): twisted rosettes; (i) and (j): lobed cotyledon. RNAi plants are T_3_ generation with from line RNAi-A3. Size bar 2 mm for b and g–i, 1 mm for a, c–f, and 3 mm for normal phenotype. (b) and (f) originally published in [5].

We previously reported an increase in phenotypic diversity of *A. thaliana* seedlings when HSP90 was pharmacologically inhibited by growth on GDA [5]. In an initial examination, we observed similar phenotypes, as well as similar phenotypic diversity, in the HSP90-RNAi lines. These phenotypes included the accumulation of purple pigment, an HSP90-dependent phenotype specific to the Col-0 accession in the previous study (Fig. 2). As *A. thaliana* is an inbreeding species[39], common laboratory accessions such as Col-0 are homozygous at virtually all nucleotides. Therefore, the phenotypic diversity revealed within the Col-0 accession by HSP90 reduction is highly unlikely to be due to genetic differences between individual plants. Rather, the diversity represents the outcome of stochastic developmental processes [5].

**Figure 3:**
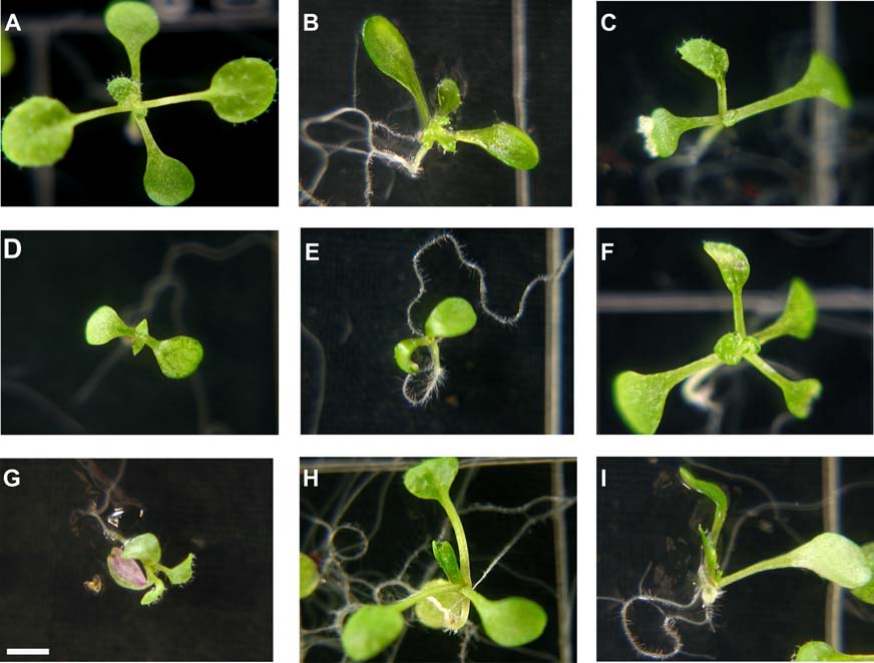
Examples of standardized seedling morphological phenotypes for quantitative assessment. (a) no scored phenotype; (b) extra leaf, concave cotyledons, aerial organs on growth medium; (c) missing leaf; (d) narrow leaves, delayed development; (e) shoot meristemless, delayed development; (f) necrotic spots on leaves; (g) purple pigmentation in cotyledons, delayed development; (h) curled hypocotyl with root extruded from medium; (i) no hypocotyl, aerial organs on growth medium, delayed development. All seedlings are ten days old. Size bar 2 mm.

As plants are exquisitely sensitive to environmental perturbations and often display apparently abnormal phenotypes even under non-stressful conditions, we set out to systematically investigate the phenotypic diversity in HSP90-reduced lines compared with appropriate controls. To reduce the likelihood that unrelated mutations arising during the transformation process might alter the observed spectrum of phenotypes, all transgenic lines were backcrossed twice to the Col-0 parent, with subsequent selection for insert homozygosity. Three hundred seventy-eight seeds for all five HSP90-RNAi and the three control lines were plated in a randomized block design such that nine seeds of each line were planted on each block of two plates containing thirty-six seeds each. Morphologically deviating phenotypes were scored after ten days according to a rubric standardized before the experiment (Fig. 3). To prevent experimenter bias, seed plating and phenotype scoring were conducted by a single scientist fully blind with regard to genotype identity.

**Figure 4:**
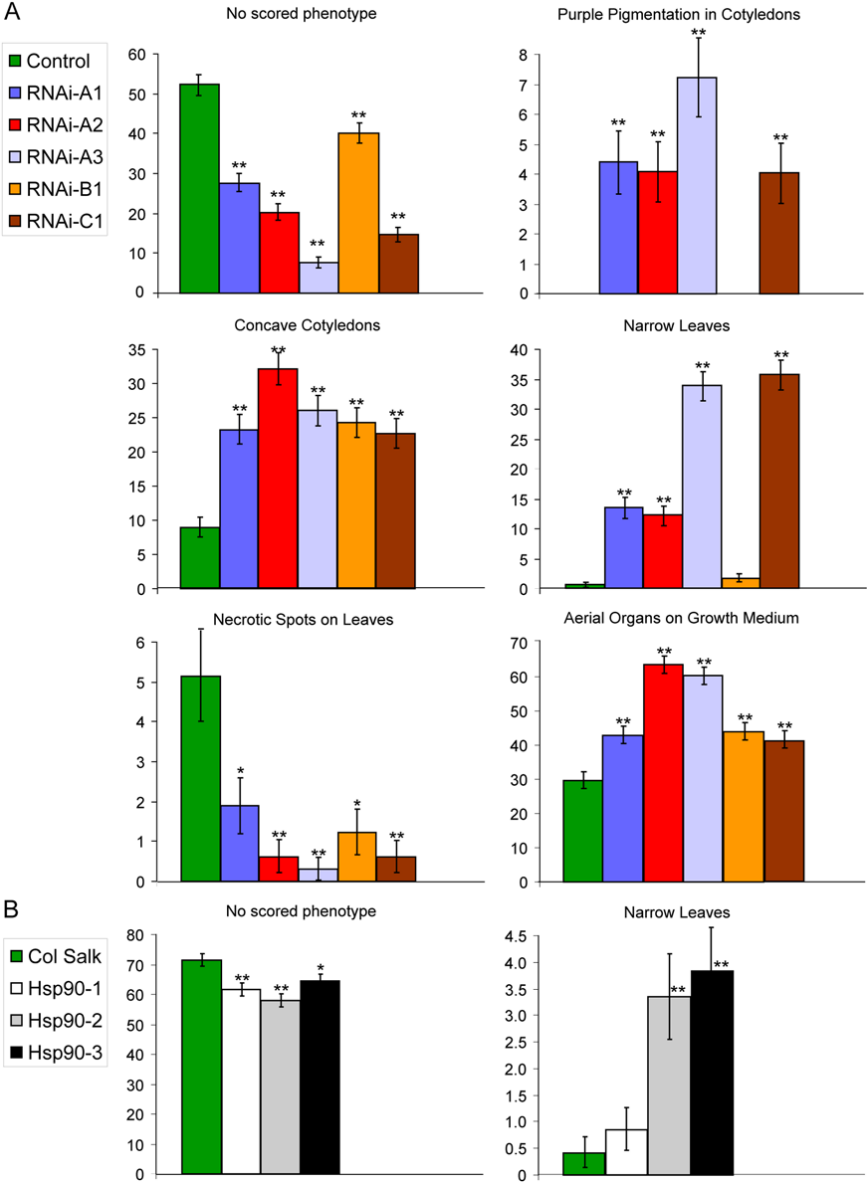
Quantitation of selected standard seedling phenotypes. (a) comparison of phenotypic frequency in percent between five HSP90-RNAi lines and three control lines (control data merged). (b) similar comparison between three HSP90-TDNA lines and Col-0. Error bars represent standard error. *p<0.01, **p<0.001; χ^2^ test.

No trait frequencies differed significantly between the three control lines; all controls were therefore combined for statistical analysis. For all five genetically HSP90-reduced lines, significantly more plants were scored as displaying at least one standardized abnormal phenotype than for the control lines (Fig. 4; p<0.0001 for each line, χ^2^ test). With the exception of RNAi-B1, all HSP90-RNAi lines showed significant enrichment for the accumulation of purple pigment in the cotyledons (p<<0.001 for each line, χ^2^ test), consistent with previous data [5]. Another cotyledon phenotype, concave cotyledons, was overrepresented in all HSP90-RNAi lines (p<<0.001 for each line, χ^2^ test). A major phenotypic difference between control and HSP90-reduced lines, excepting RNAi-B1, appeared to be the shape and timing of formation of true leaves. These HSP90-RNAi plants tended to produce narrow leaves which were often temporally delayed and therefore smaller than their counterparts on plants without the phenotype (p<<0.001 for each line, χ^2^ test); this phenotype tended to co-occur with the purple pigment accumulation (p<<0.001, χ^2^ test). No other correlations were found between other discussed phenotypes after controlling for genotype. The most common scored phenotype in both control and HSP90-RNAi plants was failure to elevate plant organs above the growth substrate; however, this phenotype was significantly more prevalent in HSP90-RNAi lines. Notably, one phenotype was significantly more prevalent in control lines: appearance of chlorotic, seemingly dead, spots on the first true leaves.

A similar experiment was conducted to assess phenotypes in the HSP90-TDNA lines. Note that as several HSP90-dependent phenotypes have previously been demonstrated to be exceptionally environmentally sensitive, phenotypic frequencies from different experiments cannot be directly compared due to potential differences in microenvironment. Importantly, aberrant phenotypes in all HSP90-TDNA lines were significantly enriched relative to the control (Fig. 4). T-DNA insertions in the *HSP90.2* and *HSP90.3* constitutive isoforms yielded a highly significant overrepresentation of seedlings with narrow leaves and delayed development (p<<0.001 for both lines, χ^2^ test), as observed with four of the HSP90-RNAi lines. Consistent with the observation that *HSP90.1* exhibits very low levels of constitutive expression [14], the HSP90.1-TDNA line was not significantly enriched for any particular scored phenotype in the non-stressful environment employed. However, significantly more plants were scored as displaying at least one abnormal phenotype (p<0.001, χ^2^ test), which may indicate that the lack of inducible HSP90 amplifies the stochastic effects of microenvironments. For a complete list of scored phenotypes, phenotype frequencies, and associated p-values, see Tables S1 and S2. In summary, a reduction in HSP90 levels results in greater phenotypic diversity, consistent with previously published hypotheses on HSP90's role in canalization [5], [40], [41].

### Enhancement of HSP90-Dependent Phenotypes by Altered Temperature

**Figure 5:**
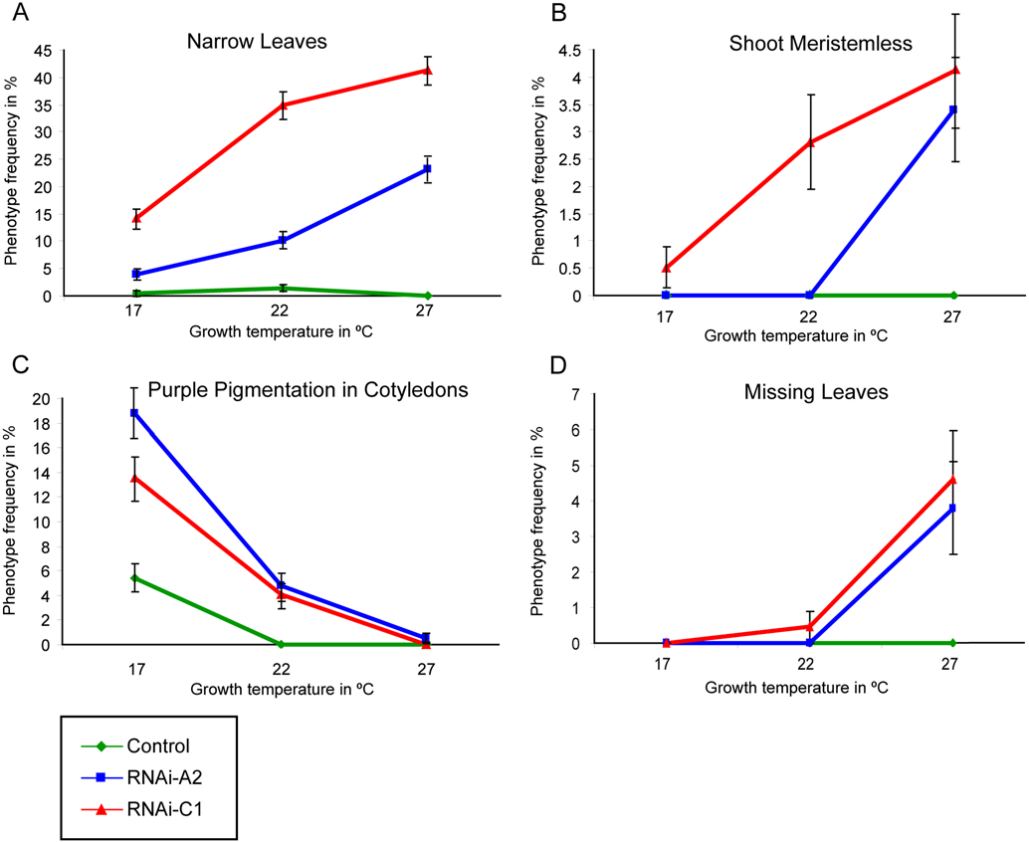
Response of selected seedling phenotypes to growth temperature. (a) narrow leaf phenotype; (b) shoot meristemless phenotype; (c) purple pigment accumulation; and (d) one of first true leaves missing. Phenotypes for two HSP90-RNAi lines and one control were measured at 17, 22, and 27°C. Error bars represent standard error.

As previous studies have described HSP90-dependent phenotypes that can be enhanced or mimicked by temperature treatment [5], [6], we investigated whether the genetically HSP90-reduced lines recapitulate this phenomenon (Table S3). We analyzed seedling phenotypes as above, but at three different growth temperatures. Since higher temperatures accelerate development, seedlings were scored at different times after germination but at comparable developmental stages. Our previous studies indicated that the narrow leaf phenotype was revealed at higher growth temperatures (27°C) in genetically predisposed lines [5]. In agreement, increased temperature significantly increased the frequency of this phenotype in predisposed HSP90-RNAi lines (p<<0.001 by logistic regression of temperature versus phenotype occurrence); no such increase was observed in the control (Fig. 5). Note that the severity of the phenotype was also dramatically enhanced, with 3.5 percent of HSP90-RNAi plants presenting as shoot meristemless at the highest temperature (p<0.001 by logistic regression).

The number of seedlings missing one of the first true leaves was also significantly enhanced by increased temperature in the HSP90-RNAi lines (p = 0.001, χ^2^ test of 22°C versus 27°C), but not in controls (Fig. 5). Significant deviations of the development of repeated characters are commonly associated with a decrease in developmental stability. Therefore, not only does a decrease in HSP90 function result in lower developmental stability, but this effect is further exacerbated by elevated temperature.

Notably, certain HSP90-dependent phenotypes are not exacerbated by increased temperature; rather, the frequency and severity decreases [6]. For example, the frequency of the accumulation of purple pigment in cotyledons is dramatically decreased at higher growth temperatures (Fig. 5; p<<0.001 by logistic regression). Indeed, even control plants significantly displayed this phenotype at the lowest tested temperature (p = 0.003, Fisher's Exact Test of 17°C versus 22°C), though at a lower frequency than HSP90-RNAi seedlings. Taken together, these data support a role for HSP90 in the genesis of plant plasticity, the maintenance of wild-type plastic responses, and the promulgation of developmental stability.

### Quantitative Phenotypic Differences in Adult HSP90-Reduced Plants

The genetically HSP90-reduced sets are ideal instruments to examine HSP90's role in the adult plant, as our previous work was limited by the light-sensitivity of GDA to the assessment of the effects of reducing HSP90 function in seedlings. To investigate phenotypes enriched in adult HSP90-reduced plants, we measured a variety of life-history traits and altered morphologies. Forty plants of each of the HSP90-RNAi, HSP90-TDNA, and appropriate control lines were grown in a randomized design under standard greenhouse conditions (see Methods).

**Figure 6:**
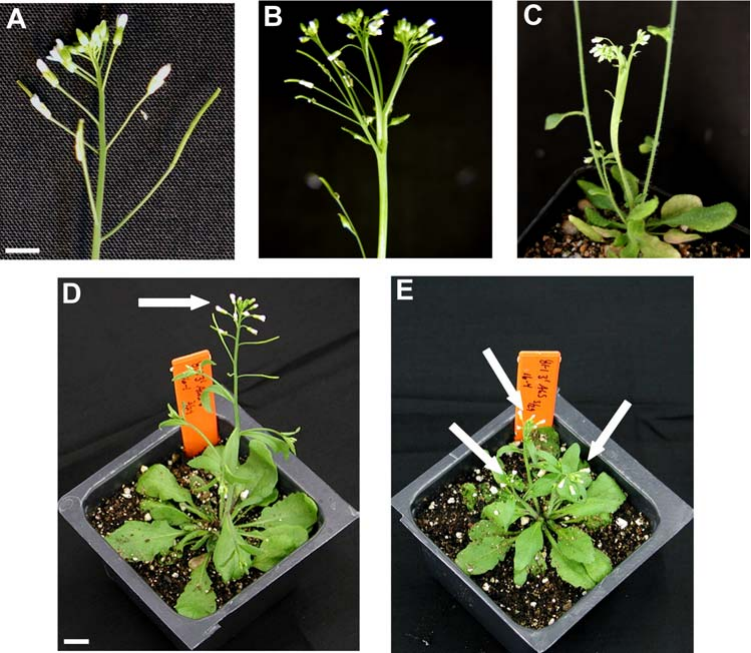
Examples of altered morphologies observed in adult HSP90-reduced plants. (a) control inflorescence; (b and c) fasciated inflorescence; (d) normal morphology with single primary inflorescence; (e) multiple simultaneous developing inflorescences. White arrows in d and e denote primary inflorescences. Scale bar 5 mm for a–c, 1 cm for d,e.

**Figure 7:**
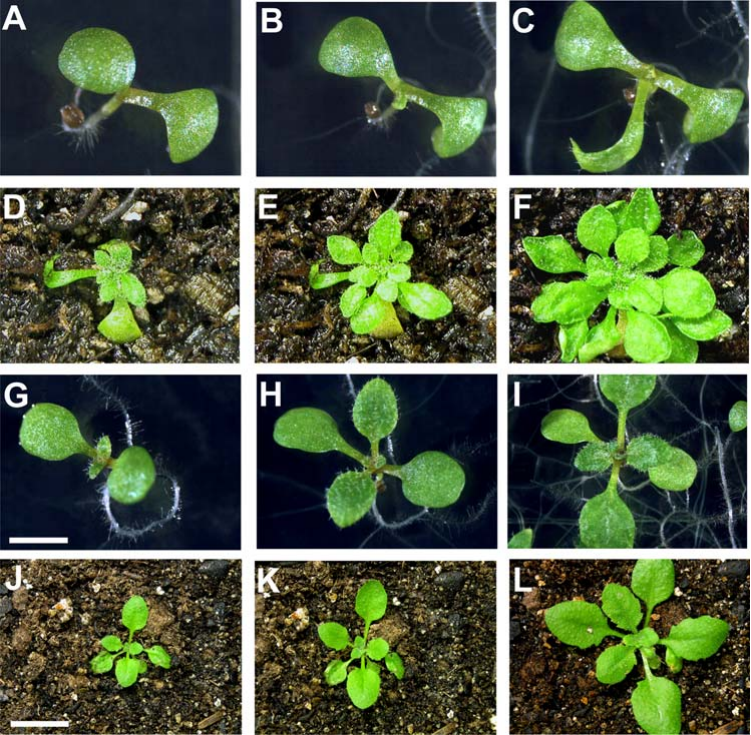
Developmental progression of common altered shoot meristem development phenotype. (a–f): Seedling with RNAi-A construct exhibiting delayed development of true leaves and shoot meristem organization defects resulting in overproliferation of leaves. (g–l): Seedling with control construct with normal morphology. (a and g) 7 days after germination; (b and h) 10 days; (c and i) 14 days; (d and j) 18 days; (e and k) 21 days; (f and l) 25 days. Scale bar 1 mm for a–c, g–h, 2 mm for i, and 1.5 cm for d–f, j–l. These seedlings are of the Shahdara ecotype and are used to illustrate the phenotype; the developmental progression of affected Col-0 seedlings is similar.

Two phenotypes affecting gross morphology were significantly enriched in HSP90-RNAi lines (Fig. 6). The most frequent was loss of apical dominance, leading to the simultaneous emergence of multiple primary inflorescences. Plants which grew multiple inflorescences as adults tended to be those which exhibited narrow first true leaves or other shoot meristem defects as seedlings (Fig. 7), indicating that the adult phenotype is likely a result of the early defect. This phenotype was observed in all five HSP90-RNAi lines (48% total frequency; p<<0.001 versus control, Fisher's Exact Test) but not in controls or in HSP90-TDNA lines.

The second abnormal morphological phenotype presented as multiple shoots which initially developed fused together but eventually separated (Fig. 6). This morphology occurred in all HSP90-RNAi lines except for RNAi-B1 (4.7% frequency in combined HSP90-RNAi lines, p = 0.0216 versus control, Fisher's Exact Test) but not in any controls or HSP90-TDNA plants. No homeotic transformations were observed in any plant.

Several quantitative life-history traits were altered in Col-0 HSP90-reduced plants (Table 2). All lines demonstrated to have constitutively lower HSP90 expression levels flowered significantly later than controls (p<0.05 for each line versus control, Tukey's HSD). As *A. thaliana* ceases to produce rosette leaves when flowering commences, leaf number is correlated with flowering time; all five HSP90-RNAi lines had a significantly greater number of leaves than the control (p<0.05 for each line versus control, Tukey's HSD). Except for line RNAi-B1, all the RNAi lines were significantly reduced in rosette diameter (p<0.05 for each line versus control, Tukey's HSD). The total weight of seeds produced by each HSP90-reduced line was lower than all control lines (p<<0.001 by ANOVA of all HSP90 reduced lines versus controls). As the average seed weight was not significantly affected by HSP90 levels, HSP90-reduced plants produce slightly fewer seeds and likely experience a small reduction in fitness in the environment tested. All of these phenotypes were affected by the presence of multiple primary inflorescences; however, all still significantly differed in HSP90-reduced plants after controlling for the altered morphology (data not shown). Therefore, the HSP90-dependent morphological and quantitative phenotypic differences do not constitute a single syndrome resulting from HSP90 reduction.

**Table 2:** Quantitation of selected life-history traits.

Genotype	Flowering Time (days)		Total Leaf Number (primary meristem)		Longest Leaf Length (mm)		Total Seed Weight (mg)	
	Mean	Tukey's HSD	Mean	Tukey's HSD	Mean	Tukey's HSD	Mean	Tukey's HSD
Control (RNAi)	27.3	B	17.8	DE	5.08	A	143	AB
RNAi-A1	28.8	A*	19.8	BC*	4.64	BC*	134	ABC
RNAi-A2	30.0	A*	22.8	A*	3.95	D*	113	E*
RNAi-A3	28.7	A*	19.7	AB*	3.94	D*	118	DE*
RNAi-B1	29.0	A*	21.6	BC*	5.01	AB	130	BCD
RNAi-C1	29.1	A*	22.8	A*	4.35	C*	129	CDE*
Control (Col-0)	26.9	B	17.5	DE	4.98	AB	146	A
*hsp90-1*	27.1	B	17.0	E	5.04	A	123	CDE*
*hsp90-2*	28.6	A*	18.9	CD	5.09	A	126	CDE*
*hsp90-3*	28.6	A*	18.3	CDE	4.99	AB	127	CDE*

Means are least-squares means (see Methods). Significance by Tukey's HSD test; lines not sharing the same letter for a trait are significantly different at α = 0.05 (*).

Several other measured phenotypes were significantly different between some HSP90-RNAi lines and the controls, including plant height, silique number, and dry aerial mass. These differences could be statistically explained by the presence of multiple primary meristems and are likely a consequence of the early morphological difference.

### Genome-Wide Expression Analyses Suggest Altered Plastic Responses in HSP90-Reduced Lines

As little is known about HSP90's functions in plants, we attempted to extract biologically relevant pathways modulated by HSP90 from the genome-wide expression analyses discussed above. Using the several hundred significantly changed genes in each line, we determined if the significant genes had an increased frequency, when compared to all genes on the array, of Gene Ontology (GO)-defined biological processes, molecular functions, and cellular components [42] with GeneMerge [43]. All enrichments and corresponding significances are included in Dataset S1.

A comparison of all HSP90-reduced lines versus all controls reveals that the environmental responsiveness of the HSP90-reduced plants is generally altered. For example, the biological processes “response to abscisic acid stimulus” (p = 0.0002; hypergeometric distribution test with multiple comparison correction), “response to water deprivation” (p = 0.0004), and “jasmonic acid biosynthetic process” (p = 0.037) were significantly enriched among upregulated genes. Similarly, the molecular function “transcription factor activity” (p = 0.008) was significantly enriched among upregulated genes, consistent with HSP90's established role in the maturation of signal transduction proteins in other organisms [2], [3].

When examining GO category enrichments in individual lines, many more significant values were found in the HSP90-RNAi lines than in the HSP90-TDNA lines, likely due to the observed heat-stress induction in the latter. The hypothesis that HSP90 functions to allow the plant to respond appropriately to environmental cues was strongly supported by the analysis of individual HSP90-RNAi lines. Enriched biological processes included responses to various hormones and abiotic and biotic stresses and stimuli. The “endomembrane system” cellular compartment category was consistently enriched in genes with significantly altered expression. Most of the genes contributing to this category enrichment were again those involved in responses to environmental stimuli. Unlike the single isoform insertion mutants, the *hsp90.2-3* mutant extensively shared the enrichments in environmental response categories observed with the HSP90-RNAi lines.

### Response to Herbivory

Previous studies have demonstrated that HSP90 inhibition results in the attenuation of several R-gene signaling pathways, leading to increased sensitivity to biotrophic pathogens, including viruses, microbial pathogens, and certain metazoan parasites [18]–[24]. R-gene-dependent defense involves salicyclic acid (SA) mediated signaling [25]. SA-mediated defense against microbial pathogens and jasmonic acid (JA)-mediated defense against insect herbivores [44] have been suggested to be antagonistically regulated [45], [46]. Our expression analysis is in agreement with this antagonistic regulation, as it revealed that plants reduced in HSP90 generally display upregulation of genes involved in jasmonic acid biosynthesis and that several lines exhibit downregulation of salicylic acid biosynthesis genes. Therefore, we used caterpillars of the cabbage looper *Trichoplusia ni* to test the basal resistance of all HSP90-reduced lines to a generalist herbivore.

Plant resistance to the herbivore was quantified by the dry weight of a single caterpillar after seven days of feeding on a single plant, as weight gain of the caterpillar is inversely correlated to plant resistance. To control for possible effects of microenvironments, all experiments were randomized with respect to plant genotype. No consistent significant change in basal resistance was observed for individual T-DNA insertion mutants or for the HSP90-RNAi lines except for RNAi-B1, which was more resistant than the control. The most significant and reproducible difference to controls was observed with the *hsp90.2-3* mutant, known to be more sensitive to biotrophic pathogens, which was significantly more resistant to herbivore attack. In this experiment, we also observed that the *hsp90.2-3* mutant plants flowered significantly later than the control in these growth conditions (data not shown), consistent with the later flowering time of other HSP90-reduced plants reported above.

**Figure 8:**
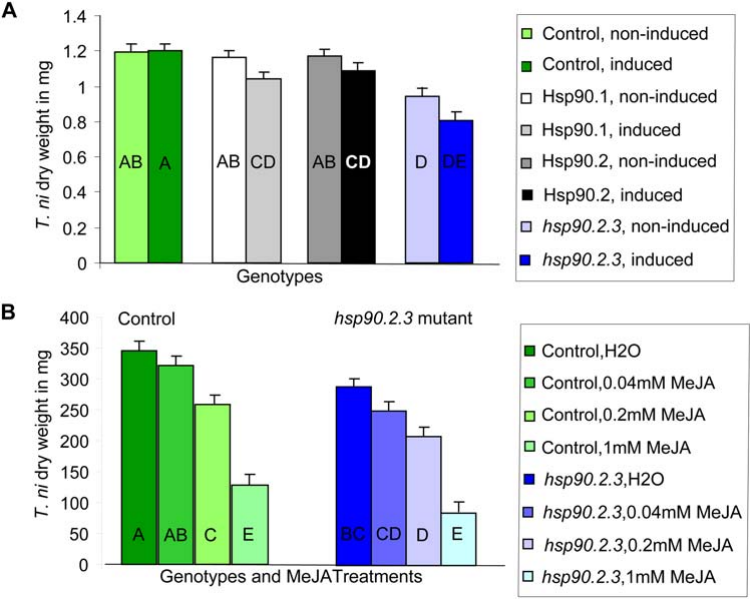
Response to herbivore attack differs in HSP90-reduced lines. Response to herbivory was measured by *T. ni* caterpillar dry weight. Least-squares means of caterpillar weight are plotted for each genotype and treatment (see Methods). Top: Basal and induced herbivore responses. For each pair, lighter colors denote basal response, darker colors indicate response after prior herbivore attack. Bottom: Herbivore response after treatment with different concentrations of Me-JA. Control in shades of green; *hsp90.2-3* in shades of blue. Lighter colors indicate higher Me-JA concentration. Statistical analysis by pairwise Student's t-tests; the response of lines sharing any letter is not statistically different. Separate statistical analysis conducted for top and bottom panels.

Previous studies have established that plants induce lasting responses to herbivores upon attack [47]. We next addressed whether the constitutively upregulated defense responses of the *hsp90.2-3* mutant are already maximal or whether they could be further induced. One three-day old *T. ni* caterpillar was caged on one standardized leaf per plant for 24 hours. Plants were allowed to recover and induce resistance for four days, after which a second *T. ni* was added as before. The *hsp90.2-3* could indeed induce further resistance (Fig. 8). This experiment also included the HSP90.1-TDNA and HSP90.2-TDNA insertion mutants, which were not significantly different from the control without induction. Interestingly, both these lines were significantly more resistant than the control after the induction treatment.

A final experiment examined whether the constitutive resistance observed in the *hsp90.2-3* mutant resulted in saturation of JA signaling. Three concentrations of methyl-JA (Me-JA) were applied to *hsp90.2-3* and control plants, followed by the *T. ni* resistance bioassay. In both genotypes, herbivore resistance increased with increasing concentrations of Me-JA. However, the *hsp90.2-3* mutant was more resistant than the control for a given concentration of Me-JA (Fig. 8). Thus, the signaling capacity of the JA pathway is not saturated in the *hsp90.2-3* mutant. The observed additive effect of genotype and treatment does not exclude the hypothesis that reductions in HSP90 capacity result in increased herbivore resistance by a JA-independent pathway. In summary, the increased sensitivity to microbial pathogens and decreased sensitivity to herbivores of the *hsp90.2-3* mutant suggest an important role for HSP90 in the integration of diverse environmental stimuli into appropriate organismal responses.

## Discussion

Our analysis of plants with genetically reduced HSP90 levels establishes that HSP90 is crucial to maintain wild-type morphology against stochastic developmental or environmental perturbation. Further, we demonstrate that HSP90 is a central regulator of the plastic responses of plants to their environment.

To determine the phenotypic consequences of reduced HSP90 function in *A. thaliana*, we employed complementary sets of HSP90-reduced lines, comparing lines with multiple cytosolic isoforms reduced via an RNAi approach with T-DNA insertion lines representing null mutants of individual isoforms. Note that the HSP90 reduction in these lines is not severe enough to interfere with plant viability or the health of most tested individuals. Therefore, these lines allow the assessment of traits which are most dependent on full HSP90 function.

To control for possible insertion site effects of the RNAi constructs, five independent HSP90-RNAi lines were analyzed, and constructs with sequence specificity to three different portions of the HSP90 isoforms were used. As HSP90-dependent phenotypes tend to be shared among the different HSP90-reduced lines and our analysis of the whole-genome expression data suggests that off-target effects are highly unlikely, we conclude that our results represent a true description of the effects of modest HSP90 reduction in *A. thaliana*. Indeed, the HSP90-RNAi set may more faithfully reveal effects of reduced HSP90, as these lines, unlike individual constitutive HSP90-TDNA lines, display neither compensation through upregulation of the inducible isoform nor mild induction of the heat-shock response. All the HSP90-reduced lines described in detail are in the Col-0 background; therefore, we do not here address the buffering of polymorphic genetic variation by HSP90 [5]. Analysis of other accessions may reveal that Col-0 is genetically predisposed to exhibit some of the revealed phenotypes upon reduction of HSP90 function.

Our comprehensive quantitative analysis of seedling phenotypes in genetically HSP90-reduced plants extended the previous qualitative study of seedlings with HSP90 partially inhibited by GDA [5]. In seedlings, both methods of HSP90 reduction resulted in similar morphological abnormalities, such as altered leaf shape, organ number, and pigment accumulation. As isogenic plant seedlings may display significant phenotypic variability even in highly controlled environments, we established a standardized set of common morphological abnormalities (Fig. 3) whose frequency was assessed across a population randomized by genotype by a scientist lacking knowledge of plant identity. Both sets of genetically reduced HSP90 lines showed a significant increase in morphological abnormalities relative to their respective controls (Fig. 4). 

These data are consistent with our previous findings that HSP90 may promote developmental robustness, such that a decrease in HSP90 function results in an increase in the frequency of stochastic developmental events, thereby amplifying phenotypic variation in an isogenic population. Developmental stability is often measured by the faithful development of repeated characters such as leaves. HSP90 reduction led to a significant increase in early leaf number defects, exacerbated by further challenge with higher temperature. Similarly, reduction of HSP90 function has also been linked to increased stochastic variation in highly canalized characters in *Drosophila melanogaster* [9].

Both sets of HSP90-reduced lines display significantly increased frequencies of abnormal shoot meristem development. These alterations display a range of temperature-dependent expressivity, from abnormally narrow leaves to complete absence of adult leaf development. Affected plants normally recover later in development; however, this initial defect commonly results in the maintenance of multiple primary shoot meristems in the adult plant.

The timing of flowering is among the most important decisions in the plant lifecycle and requires the integration of multiple environmental signals through complex signaling pathways. HSP90-reduced lines consistently flowered later than controls, suggesting that the chaperone may play a role in either sensing or integrating environmental signals into the appropriate response.

Curiously, one phenotype, necrotic spots on leaves, was more frequently observed in both sets of control lines. We speculate that, given HSP90's established role in the maintenance of R-proteins, these spots may correspond to localized cell death in response to inappropriate activation of biotroph resistance pathways. The reduced incidence of necrosis in HSP90-reduced plants is consistent with the destabilization and degradation of certain R-proteins in an HSP90-reduced background.

Such diminuation of SA-dependent defense responses suggests that plastic responses regulated in an antagonistic manner may be upregulated. Indeed, our whole-genome expression analysis revealed that genes affecting JA biosynthesis tended to be upregulated, indicating that HSP90-reduced plants may display increased resistance to insect herbivores. While basal resistance was not reproducibly affected in the HSP90-RNAi or HSP90-TDNA lines, the stronger *hsp90.2-3* mutant consistently showed greater resistance to the generalist herbivore *T. ni*. When resistance was induced by prior caterpillar herbivory, both the *hsp90.2-3* mutant as well as the tested HSP90.1-TDNA and HSP90.2-TDNA lines were more responsive to the mild induction treatment than the control. Therefore, the resistance of the *hsp90.2-3* mutant represents greater expressivity of the phenotype. Our data mirror the observed sensitivity of the *hsp90.2-3* mutant to microbial pathogens, consistent with the suggested cross-regulation between the pathogen defense and herbivore resistance pathways. Note that, as with basal herbivore resistance, basal defense against microbial pathogens was not affected in the HSP90.2 isoform mutant. Thus, HSP90 is key to maintaining the responsive plasticity of the defense system; reduction of HSP90 function predisposes the plant to herbivore defense but renders it sensitive to pathogen attack.

Our results demonstrate the involvement of HSP90 in the plastic balance of defense responses. Our genome-wide expression analysis and previous studies reporting HSP90-dependent effects on other classical plastic responses, such as the response to light and gravitropism [5], [16], suggest that HSP90's effects on plant plasticity are generalizable to many different stimuli and that HSP90 plays a global role in the environmental responsiveness of plants. The plant system, in particular with the comprehensive set of HSP90-reduced lines presented here, provides a unique opportunity to further investigate the hypothesis of HSP90 as a central interface between organism, development, and environment.

## Materials and Methods

### Construction of HSP90-Reduced Lines

DNA constructs were created following the procedures in [34]. Using the pCGN1547 vector backbone [48] modified with a cauliflower mosaic virus 35S promoter and a nopaline synthase terminator [49], fragments of HSP90 sequence were cloned in tandem antisense and sense orientation, thus forming a dsRNA hairpin upon transcription. Four different fragments of the coding sequences of HSP90 were used: bases 678-1154 of *HSP90.1* (At5g52640) (RNAi-A), bases 1572-2100 of *HSP90.1* (RNAi-B), bases 1551-1941 of *HSP90.2* (At5g56030) (RNAi-C), and bases 310-695 of *HSP90.3* (At5g56010) (RNAi-D). The coding sequence of mGFP4 [50] was inserted between the antisense and sense HSP90 sequences to improve construct stability in bacteria. Control constructs contained the GFP fragment without any HSP90 sequence. All constructs were verified by direct sequencing.

*Agrobacterium tumefaciens* strain ASE was used to transform the constructs into *A. thaliana* (Col-0, laboratory stock from D. Preuss) plants by vacuum infiltration [51]. Transformed T_1_ seedlings were selected on germination (GM) [5] medium containing kanamycin (50 µg/mL). Kanamycin resistance was assayed by root elongation after 14 days of growth. Resistant seedlings were transferred to soil and allowed to self-pollinate. At least 100 T_2_ seeds per T_1_ line were grown on kanamycin-containing medium to determine the resistance segregation ratio. Nine plants of each line exhibiting a segregation ratio consistent with an insertion at a single locus were transferred to soil and self-propagated. Resulting T_3_ seeds were assayed for full kanamycin resistance; fully resistant lines were used for further experiments. For some experiments, lines were backcrossed twice to the wild-type Col-0 parent, self-propagated, and fully kanamycin-resistant lines were re-isolated. The Shahdara (CS6180) lines shown in Fig. 7 were created in a similar manner.

Insertion sites were localized by TAIL-PCR, using degenerate primers AD2 or AD3 and PCR conditions as described in [35]. Construct specific right and left border primers were from [52]. The insert locations were: for RNAi-A2, ∼700 bp 3′ of *At2g04870*; for RNAi-A3, ∼100 bp 3′ of *At2g06200*; for RNAi-B1, ∼500 bp 5′ of *At1g42980*; for control-1, in the 3′UTR of *At4g10790*; for control-2, ∼100 bp 3′ of *At1g26330*; and for control-3, ∼1200 bp 5′ of *At1g25570*. Exact locations could not be determined for RNAi-A1 or RNAi-C1.

*A. thaliana* (Col-0) containing a T-DNA insert in either *HSP90.1* (SALK_075596), *HSP90.2* (SALK_058553), or *HSP90.3* (SALK_011160) [32] were obtained from the Arabidopsis Biological Resource Center. Lines homozygous for each insert were isolated by PCR and were verified to be true-breeding by the PCR analysis of several progeny.

### Assessment of HSP90-Reduction

For the analysis of HSP90 levels by quantitative Western blot, seeds were plated randomized with respect to genotype on GM medium. HSP90-RNAi and HSP90-TDNA lines were grown separately. The HSP90-RNAi seeds were T_3_ generation without backcrossing. Plates were cold-treated at 4°C for four days to ensure equal germination and incubated at 22°C under 100 µmol m^−2^ s^−1^ cool-white light in a 16 hour light/8 hour dark (long-day) cycle. The aerial portions of individual plants were harvested after fourteen days of growth and were immediately frozen in liquid nitrogen. The plants were individually ground to a powder in microfuge tubes, on dry ice, using disposable plastic pestles. Lysis buffer (50 mM Tris-HCl, 100 mM NaCl, 2 mM EDTA, and 1% NP-40) was added to the powder on ice and mixed by pipetting. Five plants from each line were pooled for each data point, with two biological replicates for each line. Protein concentrations were measured using the Biorad DC protein assay and were equalized by addition of buffer. Equal amounts of each line were loaded onto an SDS Page gel. A standard curve of one of the control accessions, control-1, was run on each gel. Proteins were transferred from the gels to PVDF membranes and Western blotted for HSP90 using an *A. thaliana*-specific rabbit polyclonal antibody [36]. Presence of the secondary was detected using ECL reagent and film. Bands on the film were quantitated using an Alpha Innotech Imager and its accompanying Chemi Imager Alpha Ease FC software, with experimental samples plotted on the standard curves generated by the control samples.

For the quantitation of HSP90 levels by RT-PCR, seedlings were grown on soil (Fafard #2), with 10 plants per 4” pot. The HSP90-RNAi seeds used were T_3_ and T_4_ generations without backcrossing. Aerial tissue from 14 day old plants which had been heat shocked at 37°C for two hours was harvested and flash frozen in liquid nitrogen. Plants (5−10) for individual genotypes were pooled and manually ground in liquid nitrogen. Total RNA was isolated using the SV Total RNA Isolation kit (Promega) protocol on the ground tissue, omitting the DNase treatment in the manufacturer's protocol. Ten µg of eluted total RNA was treated with DNA-free DNase Treatment (Ambion). One hundred µL of supernatant from this step was used in a second Turbo DNA-free DNase Treatment (Ambion). RNA was precipitated with NaOAC and isopropanol, washed with 75% ethanol, and resuspended in 20 µL water. For accurate quantitation, the RNA was assayed with the Quant-iT RiboGreen RNA Assay Kit from Molecular Probes (Invitrogen). After Ribogreen quantitation, 500 ng of RNA was used in a random primed first strand synthesis reaction: Five hundred ng RNA was mixed with 1 µL 250 ng/µL random primers (Invitrogen 48190-011), 1 µL dNTP's (Invitrogen 18427-013), and water for 10 µL total volume. The mixture was incubated at 65°C for five minutes, then 4°C for two minutes. Four µL first strand buffer (Invitrogen), 2 µL of 0.1M DTT, and 1 µL of water were then added to each reaction, and the mixture was incubated at 25°C for two minutes. One µL SuperScript II RNase H Reverse Transcriptase (Invitrogen 18064-014) was added, and the mixture was incubated at 25°C for ten minutes, followed by 50 minutes at 42°C, 15 minutes at 70°C, and cooled to 4°C. The resulting cDNA was labeled with the QuantiTect SYBR Green PCR Kit (Qiagen 204143) in a DNA Engine Opticon PCR cycler (MJ Research) following the kit protocol. The cDNA was amplified by primers TGGTTCTGAAAACTTCTAATATGTCG and TGACACAAACCCAACCCTAGA, which amplify a portion of the 3′ untranslated region of *HSP90.1*. The PCR cycle employed was 95°C for 15 minutes, followed by 40 cycles of 94°C for 15 seconds, 55°C for 30 seconds, and 72°C for 30 seconds, with product concentration measurement at the end of each cycle. The standard curves used in the SYBR Green RT-PCR assay were derived from genomic *A. thaliana* (Col-0) DNA, purified with DNeasy Plant Maxi Kit (Qiagen 68163). This DNA was sheared by passing it through a BD 18× 1B needle attached to a 1 mL BD slip-tip disposable tuberculin syringe. The DNA was then quantitated with a spectrophotometer, and the concentration diluted to 2.5 ng/µL. Four 10:1 serial dilutions were made from this stock. These five standards were used to generate the standard curve, with each dilution run in triplicate. All samples were also run in triplicate, along with template-only controls. Determination of cycle time values were done with the Opticon Monitor 2 analysis software.

### Assessment of Phenotypic Diversity in Seedlings

Three hundred seventy-eight seeds from each of five HSP90-RNAi and three control lines, all backcrossed, were plated on gridded square petri plates containing GM media at an equally-spaced density of 36 seeds per plate. The experiment was randomized such that nine seeds of each line were placed randomly on each block of two plates. A second experiment was performed with the three T-DNA insertion lines, along with a Col-0 control (CS60000), with 504 seeds per mutant line and randomization within each plate. All seeds used in each experiment resulted from parental plants which had been propagated in the same flat and harvested at the same time.

Plates were stratified and plants grown as for the assessment of HSP90-reduction above. Morphologically deviant phenotypes were analyzed visually after ten days of growth. At this time, most plants displayed fully developed first true leaves and had initiated spirally phyllotactic leaves. Scoring was performed according to a standard rubric developed during pilot experiments. Each line had been included in at least one pilot experiment; the data presented here are from single experiments for the HSP90-RNAi and HSP90-TDNA lines. Data from pilot experiments were consistent with those presented here. The following traits were scored (Fig. 2): missing/extra organs; aberrant cotyledon shape, color, or expansion; aberrant leaf shape or color; failure to lift apical organs off the growth medium; delayed development such that spirally phyllotactic leaves were not visible; curled hypocotyl; and failure to germinate. Plants classified as “shoot meristemless” displayed no growth of apical-meristem derived organs when viewed under a dissecting microscope at 20× magnification. Plants without any standardized phenotype were scored as “no scored phenotype”.

A similar design was employed to assess the temperature dependence of observed morphological phenotypes. One control line and two HSP90-RNAi lines (RNAi-A2 and RNAi-C1) were used, with 360 seeds per temperature per line and randomization within each plate. Plant growth conditions were as above except that temperatures of 17°C, 22°C, and 27°C were used. To account for more rapid development at increased temperature, seedlings were scored at eight days after germination (27°C), ten days (22°C) and fourteen days (17°C), when the developmental stages were equivalent. A restricted set of phenotypes was scored.

The identities of all lines were arbitrarily coded before being given to the experimenter. Therefore, all plating and scoring was performed without knowledge of the genotype of each plant.

As no phenotypes were statistically different between the three control lines for the HSP90-RNAi lines, the controls were combined for statistical analysis. To compare the frequencies of extremely rare phenotypes (less than five occurrences in either group under comparison), we used Fisher's Exact Test (2-tailed). For more common phenotypes, a 2-tailed Yates-corrected χ^2^ test was employed.

### Assessment of Phenotypic Diversity in Adult Plants

We utilized the same seed batch of the 12 lines discussed in the assessment of phenotypic diversity in seedlings. The experiment was randomized such that each line occupied eight pots in a block of three flats with 32 pots per flat; five blocks were employed for a total of forty pots per line. Four to five seeds were planted in each pot in thoroughly wetted 50% Metro-Mix 200:50% Pro-Mix BX soil. The seeds were stratified on soil for four days at 4°C, followed by growth in a greenhouse environment (∼22°C with 100 µmol m^−2^ s^−1^ cool-white light in a long-day light cycle). Flat position was rotated daily to control for microenvironments within the greenhouse. Humidity domes were removed after ten days, and all seedlings were eliminated from each pot except for the one closest to the center. No germination defects were observed. Plants were watered as necessary until one week after the cessation of flowering and were then allowed to dry for at least a month before harvest.

Flowering was defined as the date the primary bolt reached 2 cm in length. Longest leaf measurements were performed one week after flowering. Plant height and the number of siliques on the primary stem were measured at the cessation of flowering; the longest stem was used for plants with multiple primary stems. Seeds were harvested before the dry mass of above-ground tissues was measured. The mass of a counted number of seeds (∼200 per line) was measured to determine average seed mass.

No statistical differences were observed between the control lines for the HSP90-RNAi plants; therefore, these lines were combined for analysis. Multiple linear regression was used to estimate least-squares means of each trait for each line. Genotype, flat, and position within the flat (corner, edge, or interior) were treated as fixed variables. A second model adding the presence of multiple meristems as a fixed variable was created for each trait to test if observed genotypic differences could be explained by this morphological trait. Tukey's HSD post-hoc test was used to examine whether differences between lines were significant. The significance of primary shoot number distributions was tested with Wilcoxon tests.

### Genome-wide Expression Analysis

The same seed batches were used as for the quantitative Western blots, and seedlings were grown under the same conditions. Aerial tissue was harvested from 14 day old seedlings and flash frozen in liquid nitrogen. Ten plants were pooled for each genotype and manually ground in liquid nitrogen. Total RNA was isolated using the SV Total RNA Isolation kit (Promega) protocol on the ground tissue, omitting the DNase treatment in the manufacturer's protocol. Eluted total RNA was treated with RQ1 Rnase-free DNase (Promega). A 65°C, 15 min incubation step inactivated the enzyme, instead of the kit's Stop Solution. Multiple identical RNA isolations were pooled and concentrated with the RNeasy (Qiagen) cleanup protocol. 50–60 µL of solution was eluted, with 8 µg of the purified RNA was used in the standard Affymetrix cRNA labeling and hybridization protocols (http://www.affymetrix.com/support/technical/manual/expression_manual.affx) on Affymetrix ATH-1 microarrays. Three differerent experiments were performed. One experiment included lines RNAi-A1, RNAi-A3, and Control-2, with two biological replicates and one technical replicate per line. The second included lines RNAi-A2, RNAi-B1, RNAi-C1, Control-1, and Control-3, with three biological replicates per line. The third included the three single-isoform T-DNA insertion lines, along with the Col-0 control, with three biological replicates per line. All array data have been submitted to the Gene Expression Omnibus with accession number GSE7796.

All data from different experiments were pooled for preprocessing and gene expression index calculation using the Probe Logarithmic Intensity Error (PLIER) algorithm (http://www.affymetrix.com/support/technical/technotes/plier_technote.pdf). Probe sets that were below detection range across all the samples were excluded before statistical comparison. Average expression for each line was calculated by averaging across technical replicates, then averaging across biological replicates. Statistical significance was calculated by a two-way ANOVA or by Welch's t-test, depending on whether the experiment contained technical replicates. Fold changes were calculated from the average expression for each line divided by the average expression in all controls used in the same experiment. Fold changes for combined HSP90-RNAi, HSP90-TDNA, and all HSP90-reduced lines were calculated by averaging single-line fold changes converted to a linear scale.

For the analysis with GeneMerge[43], all genes with significance less than α = 0.05 were discarded. Genes were then sorted by average fold change. Lists of genes significantly upregulated by 1.5 fold or 2 fold or downregulated by 1.5 fold or 2 fold for each HSP90-reduced line were submitted to GeneMerge to determine enrichment in biological process, cellular component, or molecular function GO annotations.

### Response to Herbivory

Seeds were planted in 36-pot flats randomized with respect to genotype in thoroughly wetted Jiffy peat pellets. Three seeds were planted per pellet and were stratified for four days at 4°C. Flats were sealed with domes and parafilm to maintain high humidity during germination. Plants were grown in 12 hour light/12 hour dark cycles at ∼22°C under cool white light. Domes were removed after six days, weeded to a single plant per pot after eleven days, and fertilized with one-quarter strength Hoagland's solution [53] after fourteen days. The order of the flats was rotated daily to minimize the effects of local environmental variance.

*Trichoplusia ni* eggs were obtained from a highly inbred population (Entopath, Easton, PA) and were incubated at 28°C for two days to hatch. For the *T. ni* bioassays, the neonate larvae were caged to feed freely on single whole four-week old non-flowering plants for seven days according to [46]. Subsequently, caterpillars were removed, lyophilized, dried and weighed. Initial larval dry weight (∼10 µg) is insignificant relative to weight gain and was disregarded. For the induction experiments, at least 90 plants were used per genotype and treatment. For the induction treatment, one three-day-old *T. ni* larva was caged on the seventh leaf to feed for 24 hours. The leaf was subsequently removed and a bioassay caterpillar added as above. The same leaf was caged and removed on control plants which had no induction caterpillar.

Methyl-jasmonic acid (MeJA) was dissolved in water at three different concentrations (0.04mM, 0.20mM, 1mM) and applied to plants by aerosol spray. The plants were allowed to induce responses for two days, followed by addition of a bioassay caterpillar as above. Control plants were sprayed with water alone and were physically separated from MeJA treated plants only during spraying and induction to prevent cross-contamination. Thirty-six plants per genotype per treatment were used.

Caterpillar weight was approximately normally distributed. Least-squares means for caterpillar weight on each genotype were estimated with multiple-linear regression model including flat, treatment, genotype, and genotype×treatment interaction as fixed effects. Student's t-tests were employed to test for significant differences between genotypes.

## Supporting Information

Table S1 Morphological phenotype frequencies and associated p-values for seedling phenotypes in HSP90-RNAi lines. Top: P-values. Fisher's exact tests used for small sample sizes, otherwise chi-square tests; all tests are 2-tailed. * denotes comparisons which are not meaningful due to extremely rare observance of the phenotype (less than 0.8 percent in both lines under comparison). Bottom: Fraction of plants observed with each defined phenotype.
(0.00 MB TXT)

Table S2 Morphological phenotype frequencies and associated p-values for seedling phenotypes in HSP90-Salk lines. Top: P-values. Fisher's exact tests used for small sample sizes, otherwise chi-square tests; all tests are 2-tailed. * denotes comparisons which are not meaningful due to extremely rare observance of the phenotype (less than 0.8 percent in both lines under comparison). Bottom: Fraction of plants observed with each defined phenotype.
(0.00 MB TXT)

Table S3 Morphological phenotype frequencies and associated p-values for seedling phenotypes in HSP90-RNAi lines at different growth temperatures. Top: P-values. Fisher's exact tests used for small sample sizes, otherwise chi-square tests; all tests are 2-tailed. * denotes comparisons which are not meaningful due to extremely rare observance of the phenotype (less than 0.8 percent in both lines under comparison). Temperature effect p-values by logistic regression of temperature as a continuous variable versus phenotype presence as a nominal variable. ** denotes comparisons for which the logistic regression was not valid for the full temperature range. Bottom: Fraction of plants observed with each defined phenotype.
(0.00 MB TXT)

Dataset S1 Gene-Ontology Analysis. Significance was determined for all genes on the ATH-1 array by ANOVA comparison of normalized data from all control arrays versus all arrays from one HSP90-reduced line. All genes with p-value less significant that 0.05 (uncorrected for multiple comparisons) were discarded. Genes were then sorted by average fold change. Genes upregulated by 1.5 fold/2 fold or downregulated by 1.5 fold/2 fold were submitted to GeneMerge (http://www.oeb.harvard.edu/hartl/lab/publications/GeneMerge/GeneMerge.html) to determine enrichment in biological process, cellular component, or molecular function GO annotations. Fold changes for combined HSP90-RNAi, HSP90-Salk, and all HSP90-reduced sets were calculated by averaging fold changes converted to a linear scale. The following tables are in the format: Line name Fold Change GO Dataset GeneMerge header GO descriptor Fraction of genes with GO descriptor on ATH1 array Fraction of genes with GO descriptor significantly changed by fold change Raw e-score E-score corrected for multiple comparisons GO annotation Genes significantly changed with GO annotation
(0.80 MB TXT)
